# Structure–Function Studies and Mechanism of Action of Snake Venom L-Amino Acid Oxidases

**DOI:** 10.3389/fphar.2020.00110

**Published:** 2020-02-25

**Authors:** Anwar Ullah

**Affiliations:** Department of Biosciences, COMSATS University Islamabad, Islamabad, Pakistan

**Keywords:** snake venom L-amino acid oxidases, sequence and three-dimensional structure analysis, structure-based mechanism of action, inhibition and substrate specificity, L-amino acid oxidases and membrane interaction

## Abstract

Snake venom L-amino acid oxidases (SV-LAAOs) are the least studied venom enzymes. These enzymes catalyze the stereospecific oxidation of an L-amino acid to their corresponding α-keto acid with the liberation of hydrogen peroxide (H_2_O_2_) and ammonia (NH_3_). They display various pathological and physiological activities including induction of apoptosis, edema, platelet aggregation/inhibition, hemorrhagic, and anticoagulant activities. They also show antibacterial, antiviral and leishmanicidal activity and have been used as therapeutic agents in some disease conditions like cancer and anti-HIV drugs. Although the crystal structures of six SV-LAAOs are present in the Protein Data Bank (PDB), there is no single article that describes all of them in particular. To better understand their structural properties and correlate it with their function, the current work describes structure characterization, structure-based mechanism of catalysis, inhibition and substrate specificity of SV-LAAOs. Sequence analysis indicates a high sequence identity (>84%) among SV-LAAOs, comparatively lower sequence identity with Pig kidney D-amino acid oxidase (<50%) and very low sequence identity (<24%) with bacterial LAAOs, Fugal (L-lysine oxidase), and *Zea mays* Polyamine oxidase (PAAO). The three-dimensional structure of these enzymes are composed of three-domains, a FAD-binding domain, a substrate-binding domain and a helical domain. The sequence and structural analysis indicate that the amino acid residues in the loops vary in length and composition due to which the surface charge distribution also varies that may impart variable substrate specificity to these enzymes. The active site cavity volume and its average depth also vary in these enzymes. The inhibition of these enzymes by synthetic inhibitors will lead to the production of more potent antivenoms against snakebite envenomation.

## Introduction

Snake venom LAAO (LAAOs, EC 1.4.3.2) is an FAD-containing dimeric enzyme that stereospecifically deaminates an L-amino acid to an α-keto acid with the concomitant production of hydrogen peroxide and ammonia (Pawelek et al., 2000; Du and Clemetson, 2002; Moustafa et al., 2006; Ullah et al., 2012b; Ullah et al., 2014; Costal-Oliveira et al., 2019). These enzymes are widely distributed in the snake venom and have been found to be toxic (Li et al., 1994; Torii et al., 1997).

Upon snakebite envenomation these enzymes causes many physiological and pathological activities including induction of apoptosis (Torii et al., 1997; Ali et al., 2000; Zainal Abidin et al., 2018; Costal-Oliveira et al., 2019; Machado et al., 2019), edema (Stábeli et al., 2007; Lazo et al., 2017), platelet aggregation/inhibition (Li et al., 1994; Takatsuka et al., 2001; Sakurai et al., 2001; Stabeli et al., 2004; Izidoro et al., 2006; Toyama et al., 2006), hemorrhagic (Souza et al., 1999; Alves et al., 2008), and anticoagulant activities (Sakurai et al., 2003; Tonismagi et al., 2006). Costal-Oliveira et al. (2019) has demonstrated that LAAO from *Bothrops atrox* snake venom causes autophagy, apoptosis and necrosis in normal human keratinocytes. They also display antibacterial (Stiles et al., 1991; Stabeli et al., 2004; Toyama et al., 2006; Tonismagi et al., 2006; Stábeli et al., 2007; Abdelkafi-Koubaa et al., 2016; Rey-Suárez et al., 2018), antiviral (Zhang et al., 2003) antifungal (Costa Torres et al., 2010; Cheng et al., 2012) and leishmanicidal activity (Fernandez-Gomez et al., 1994; Tempone et al., 2001; Toyama et al., 2006; Izidoro et al., 2006; Wiezel et al., 2019).

These enzymes have anti-cancer (Sun et al., 2003; Lee et al., 2014; Tássia et al., 2017) and anti-HIV activity (Sant'Ana et al., 2008) and may be used as therapeutic agents in many disease conditions like anti-cancer and anti-HIV drugs (Sakurai et al., 2003; Zhang et al., 2004; Teixeira et al., 2016; Tan et al., 2017; Costa et al., 2017; Salama et al., 2018; Tan et al., 2018) (Sun et al., 2003; Zhang and Wei, 2007; Lee et al., 2014; Costa et al., 2014; Tássia et al., 2017; Costa et al., 2017). Besides snake venom, LAAO has been found in the insects, fungi (Nuutinen and Timonen, 2008; Yang et al., 2009; Žun et al., 2017), green algae (Schriek et al., 2009), bacteria (Arima et al., 2009), plants (Nishizawa et al., 2005) and mammals (Blanchard et al., 1944; Du and Clemetson, 2002; Kasai et al., 2010). The yellow color of most of the crude venom is due to the presence of LAAO (Tempone et al., 2001; Stábeli et al., 2007) that contains oxidized flavin adenine dinucleotide (FAD) in their structure (Pawelek et al., 2000; Moustafa et al., 2006).

LAAO is a glycoprotein with molecular mass ranging from 120–150 kDa in native (dimeric) form and 55–66 kDa in the denatured (monomeric form) (Tan and Saifuddin, 1989; Abe et al., 1998). Some reports have also shown their tetrameric existence (Georgieva et al., 2011; Feliciano et al., 2017), however, SV-LAAO is mostly present as a dimer in the solution and it is active in this state (Moustafa et al., 2006; Ullah et al., 2012b). The p*I* of these enzymes ranges from 4.4 to 8.0 (Tan, 1998). Most of the SV-LAAOs are stable when kept at room temperature (25°C) and 4°C, however, exposure to the low-temperature (–5°C and –60°C) for long period inactivates these enzymes (Curti et al., 1968; Tan, 1998). The inactivation is caused by a change in the three-dimensional structure of LAAO particularly around the active site (Soltysik et al., 1987). Interestingly, LAAOs from *Ophiophagus hannah* and *Calloselasma rhodostoma* are not inactivated by low temperature treatment (Tan, 1998).

Currently, the crystal structures of six LAAOs have been deposited to the PDB (Zhang et al., 2004; Moustafa et al., 2006; Georgieva et al., 2011; Ullah et al., 2012b; Feliciano et al., 2017). They all share the same structural fold which contains three domains: a FAD-binding domain, a substrate-binding domain and a helical domain (Moustafa et al, 2006; Georgieva et al., 2011; Ullah et al., 2012a; Zhang et al., 2004; Feliciano et al., 2017). SV-LAAOs are usually glycosylated and contain about 3–4% carbohydrates in their structure (deKok and Rawitch (1969); Hayes and Wellner, 1969) and in some cases, the carbohydrate contents may be up to 12% of the total molecular mass of the protein (Alves et al., 2008).

These enzymes hydrolyze the substrate through an oxidation-reduction reaction in which His223 act as a base abstracting a proton from the substrate (amino acid) and converting it to an imino acid (Pawelek et al., 2000; Moustafa et al., 2006). In the next step, the FAD is reduced by transferring a proton from His223. The reoxidation of FAD occurs with the addition of electrons from the oxygen. The imino acid is converted to a α-keto acid with the production of hydrogen peroxide and ammonia (Pawelek et al., 2000; Moustafa et al., 2006).

Although the crystal structures of six SV-LAAOs have been determined, no article describes all of these with comprehensive details. The current work describes the three-dimensional structural features of SV-LAAOs with special reference to their structure-based substrate specificity, mechanism of action and inhibition.

## Results and Discussion

### Sequence Alignment Analysis

**Figure 1:**
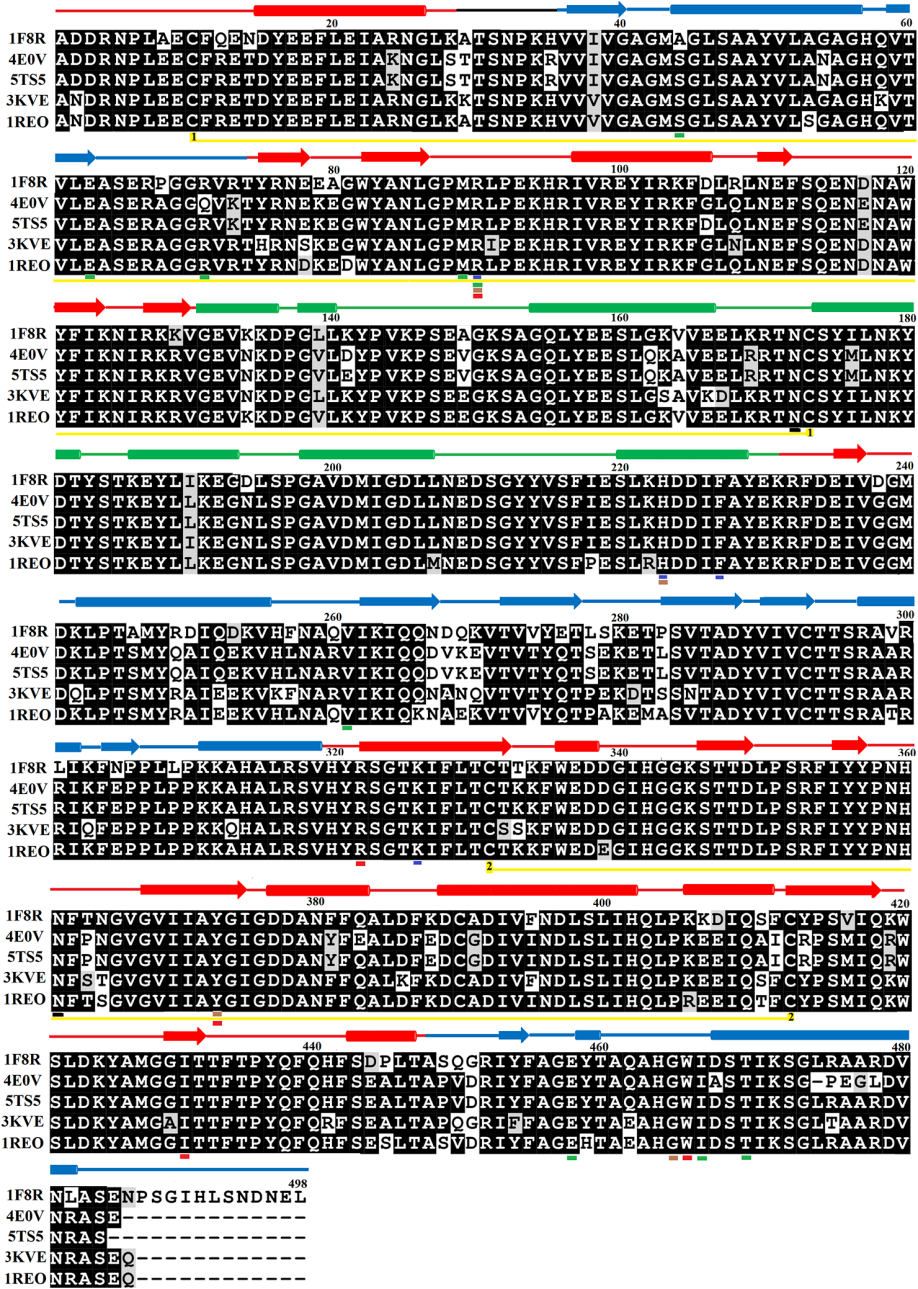
Sequences alignment among snake venom L-amino acid oxidases. 1F8R; crystal structure of L-amino acid oxidase from *Calloselasma rhodostoma*, 4E0V; Structure of L-amino acid oxidase from the *Bothrops jararacussu* venom, 5TS5; Crystal structure of L-amino acid oxidase from *Bothrops atrox*, 3KVE: Structure of native L-amino acid oxidase from *Vipera ammodytes ammodytes*, 1REO; L-amino acid oxidase from *Agkistrodon halys pallas*. The amino acid residues involved in catalysis, metal ion binding and amino acid (substrate) recognition are underlined with blue, brown, and red respectively. The FAD-binding residues are underlined in green. The cysteine residues which make disulfide bridges are linked (yellow lines). The putative N-glycosylation amino acid residues are underlined in black. The amino acid residues in FAD-binding, substrate-binding and helical domain, are colored in blue, red, and green, respectively. The secondary structure elements (alpha helices and beta strands) are shown above the sequence.

The primary structure of SV-LAAOs contains 503–516 amino acid residues in the precursor or zymogen form and 485–498 amino acid residues in the mature form (Takatsuka et al., 2001). The amino acid sequence alignment analysis indicates a high degree of sequence identity among SV-LAAOs (>84%), relatively moderate identity with Pig kidney D-amino acid oxidase (DAAOs) (~50%) and very low identity (<24%) with the bacterial (LAAO), fugal (L-lysine oxidase), and *Zea mays* Polyamine oxidase (PAAO) (**Figures 1** and **S1** and **Table 1**). The average sequence identities among SV-LAAOs and LAAOs/PAO/DAAO from other organisms are 86.80 and 31.29% respectively. The differences in amino acid residues are mostly confined to N- and C-termini in SV-LAAOs (**Figure 1**). The amino acid residues belonging to the active site (Arg90, His223, Phe227 and Lys326), FAD-binding (Ser44/Ala44 (1F8R), Glu63, Arg71/Gln71 (4E0V), Met89, Arg90, Glu457, Ileu457 and Thr469) and the substrate/ligand binding (Arg90, Tyr372 and Gly464) are fully conserved among the aligned SV-LAAOs except for Ser44 in *C. rhodostoma* and *Bothrops jararacussu* LAAOs where these have been substituted by Ala44 and Gln71 respectively (**Figures 1** and **S1**). The Cysteine residues (Cys10, Cys173, Cys293, Cys331, Cys390, and Cys413) are fully conserved in all SV-LAAOs and make two disulfide bridges (Cys10-Cys173 and Cys331-Cys413), while Cys293 and Cys390 don't form any disulfide bridge. The glycosylation sites (Asn172 and Asn361) are also conserved. The C-terminal of LAAO from *C. rhodostoma* has twelve amino acid residues more (extension) than all the other aligned SV-LAAOs sequence.

**Table 1:** Percent sequence identity among snake venom LAAOs, bacterial (L-Glutamate Oxidase), *Zea mays* (polyamine oxidase), Fungi (L-lysine oxidase) and pig kidney (D-amino acid oxidase).

Proteins	1F8R	4E0V	5TS5	3KVE	1REO	2E1M	2JAE	1B37	3X0V	1KIF
1F8R	–	84.74	86.57	86.60	88.27	34.21	26.60	25.24	24.60	45.83
4E0V	84.74	–	97.93	86.39	87.84	23.47	26.24	24.07	25.20	48.00
5TS5	86.57	97.93	–	87.60	89.26	24.38	26.42	24.38	25.25	48.00
3KVE	86.60	86.39	87.60	–	89.30	33.10	26.15	24.12	25.91	50.00
1REO	88.27	87.84	89.26	89.30	–	35.17	26.71	25.15	25.30	50.00
2E1M	34.21	23.47	24.38	33.10	35.17	–	26.67	45.00	33.11	32.00
2JAE	26.60	26.24	26.42	26.15	26.71	26.67	–	19.55	21.58	37.50
1B37	25.24	24.07	24.38	24.12	25.15	45.00	19.55	–	36.17	50.00
3X0V	24.60	25.20	25.25	25.91	25.30	33.11	21.58	36.17	–	40.00
1KIF	45.83	48.00	48.00	50.00	50.00	32.00	37.50	50.00	40.00	–

1F8R: Calloselasma rhodostoma LAAO, 4E0V: Bothrops jararacussu LAAO, 5TS5: Bothrops atrox LAAO, 3KVE: Vipera ammodytes ammodytes LAAO, 1REO: Agkistrodon halys pallas LAAO, 2E1M: Streptomyces sp. L-Glutamate Oxidase, 2JAE: R. opacus L-amino acid oxidase, 1B37: Zea mays polyamine oxidase, 3X0V: Hypocrea rufa L-lysine oxidase, 1KIF: Pig kidney D-amino acid oxidase.

The Sequence logo generated from multiple sequence alignment of SV-LAAOs indicates that the amino acid residues around the active sites and FAD-binding site are highly conserved among all the aligned enzymes (**Figure S2**).

### Overall Structure

SV-LAAO belongs to the family of enzymes called NAD(P)/FAD-dependent oxidoreductase that also comprises polyamine oxidase (PAO), flavin-containing monoamine oxidases (MAOs), D-amino acid dehydrogenase, and linoleic acid isomerase (CDD/SPARCLE; Marchler-Bauer et al., 2017).

**Figure 2:**
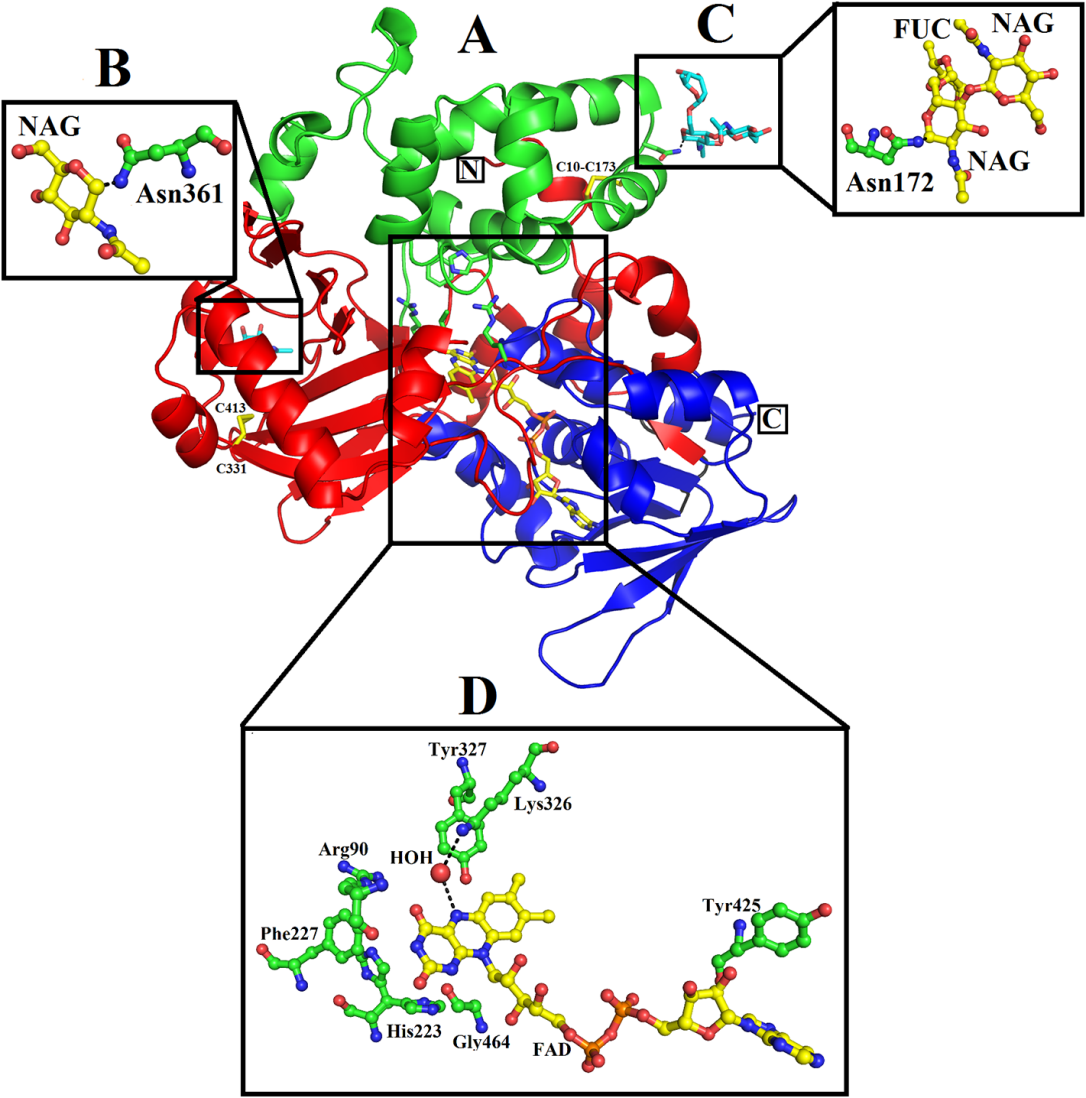
Overall structure of SV-LAAO (PDB ID: 1F8R; *Calloselasma rhodostoma* LAAO) **(A)** cartoon representation. The active site, FAD-binding and glycosylation amino acid residues are shown as green sticks. *T*he disulfide bridges are represented by yellow sticks. **(B–D)**, residues involved in glycosylation and FAD-binding highlighted. The parts of the secondary structure belonging to FAD-binding, substrate-binding and helical domains are colored in blue, red, and green, respectively.

The mature protein of SV-LAAO contains 486 amino acid residues that fold into a multidomain protein comprising of three distinct domains namely: a FAD-binding domain, a substrate-binding domain and a helical domain (**Figures 2A–D**) (Moustafa et al, 2006; Georgieva et al., 2011; Ullah et al., 2012a; Zhang et al., 2004; Feliciano et al., 2017). The overall three-dimensional structure of LAAO is composed of seventeen alpha-helices, twenty-two beta-strands and many loops that fold into three well-defined domains. The domains architecture of SV-LAAO is briefly described below:

### FAD-Binding Domain

**Figure 3:**
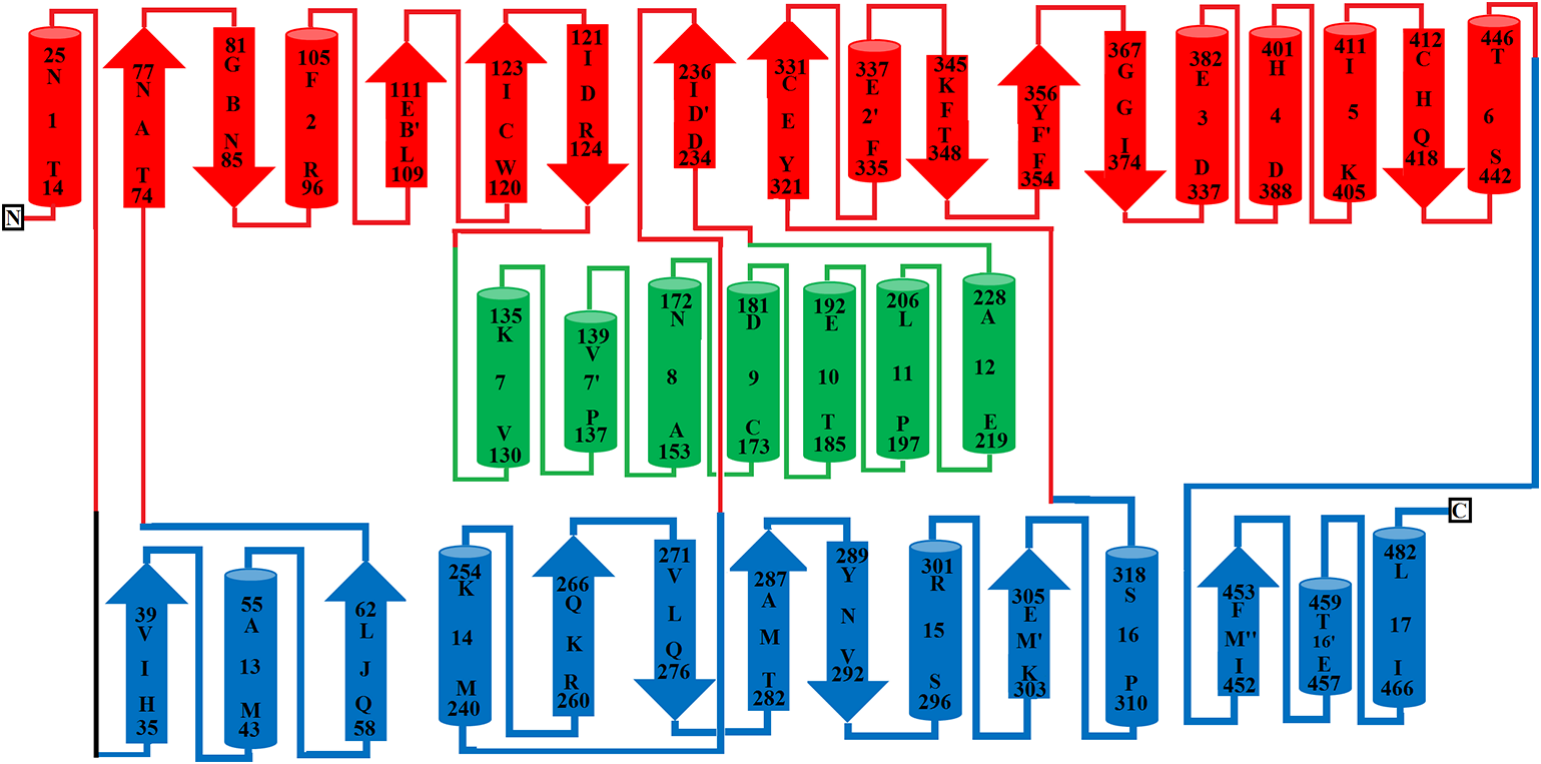
Topology diagram of SV-LAAO. The alpha helices (numbered 1–17) and beta strands (named A–N) are represented as cylinders and arrows, respectively. The short alpha helices and beta strands are shown with primes. The secondary structures and the amino acid residues in alpha helices and beta strands were assigned using the program DSSP from the primary sequence and were confirmed by PyMOL from the tertiary structure. The parts of the secondary structure belonging to FAD-binding, substrate-binding and helical domains are colored in blue, red, and green respectively.

The FAD-binding domain is composed of amino acid residues 35–72, 240–318 and 446–486 (**Figures 1**, **2A**, and **3**, **Table 3**). The secondary structure of this domain contains six beta-strands and five alpha-helices with the insertion of additional short beta-strands (two) and alpha-helix (one). Of the six beta-strands, four are parallel and two are antiparallel, while the two short beta-strands are parallel to one another. The consensus sequence of glycine residues (G40XG42XXG45) present in this domain gives close access to the negatively charged phosphate group of the cofactor and stabilizes the charge by the helix dipole. This domain is stabilized by five salt bridges that exist between the amino acid residues within this domain (Arg71-Glu457, Lys270-Asp288), and with the amino acid residues from the substrate-binding domain (Lys471-Glu13, Arg478-Glu18, Arg478-Asp15) (Sarakatsannis and Duan, 2005) (**Table 2**).

**Table 2:** Salt bridges in SV-LAAO. NH1 and NH2: Nitrogen atoms (amino groups) of the arginine side chain, OD1, and OD2: Oxygen atoms of aspartic acid side chains, OE: Oxygen atoms of glutamic acid side chains, NZ: Nitrogen atoms (amino groups) of lysine side chains.

Residue 1	Residue 2	Distance
NH1 ARG A 71	OE1 GLU A 457	2.88
NH2 ARG A 71	OE1 GLU A 457	3.58
NH1 ARG A 73	OD1 ASP A 423	3.04
NH2 ARG A 73	OD1 ASP A 423	3.19
NH1 ARG A 76	OD1 ASP A 241	2.82
NH1 ARG A 76	OD2 ASP A 241	2.73
NH2 ARG A 76	OD1 ASP A 241	2.80
NH1 ARG A 99	OD2 ASP A 234	3.63
NH2 ARG A 99	OD2 ASP A 234	2.77
NH1 ARG A 103	OE1 GLU A 100	3.13
NZ LYS A 134	OD1 ASP A 117	2.87
NZ LYS A 134	OD2 ASP A 117	3.82
NZ LYS A 151	OE1 GLU A 159	2.88
NZ LYS A 179	OE1 GLU A 167	3.86
NH2 ARG A 232	OD1 ASP A 234	2.84
NZ LYS A 270	OD1 ASP A 288	2.64
NZ LYS A 270	OD2 ASP A 288	3.82
NZ LYS A 334	OE1 GLU A 337	3.98
NH2 ARG A 353	OD2 ASP A 377	3.76
ND1 HIS A 360	OE1 GLU A 235	3.57
ND1 HIS A 401	OD2 ASP A 339	2.87
NZ LYS A 405	OD1 ASP A 391	2.68
NZ LYS A 405	OD2 ASP A 391	3.81
NZ LYS A 471	OE1 GLU A 13	2.96
NH1 ARG A 478	OE1 GLU A 18	2.60
NH1 ARG A 478	OD2 ASP A 15	3.33

### Substrate-Binding Domain

The substrate-binding domain is composed of amino acid residues 5–25, 73–129, 233–236, and 323–420 (**Figures 1**, **2B**, and **3**, **Table 3**). It contains six alpha-helices and eleven beta-strands (**Figure 3**). This domain is stabilized by an intrachain disulfide bridge (Cys331-Cys412) and an interchain disulfide bridge (Cys10-Cys173) with further stabilization by salt bridges (Lys471-Glu13, Glu18-Arg478, Asp15-Arg478, Arg71-Glu457, Arg73-Glu457, Arg99-Asp234, Arg103-Glu100, Lys334-Glu337, Arg353-Asp377, Lys405-Asp391). It also contains an N-linked N-acetylglucosamine.

### Helical Domain

This domain is continuous in the amino acid sequence and comprises of amino acids residues 130-230 and is located in between FAD-binding and substrate-binding domain (**Figure 3**, **Table 3**). The secondary structure of this domain contains six alpha-helices with one short alpha-helix and many loops. It is stabilized by an interchain disulfide bridges with the substrate-binding domain (Cys10-Cys173) and intrachain slat bridges (Lys134-Asp117, Lys151-Glu159, Lys179-Glu167).

**Table 3:** Domains of SV-LAAO.

Domains	Amino acid residues range	Total amino acid residues
FAD-Binding domain	35–64, 242–318, 446–471	130
Substrate-binding domain	5–25, 73–129, 233–236, 323–420	176
Helical domain	130–230	100

The Threading-based Protein Domain Prediction online web server identifies seven discontinuous regions from the primary amino acid sequence of SV-LAAOs belonging to these domains. The analysis indicates that these three domains are highly conserved in all SV-LAAOs and with the others proteins containing the similar structure folds in the Protein Data Bank (PDB) (**Figure S3**) (Xue et al., 2013).

The N-terminal of LAAO is stabilized by a hydrogen bond formed between Asn5 (FAD-binding domain) and Asp225 (helical domain) and the C-terminal Ser484 and His57.

### Active Site

A funnel-shaped channel is formed between the helical and substrate binding domain that starts from the surface of the protein and extends towards the active site providing access of substrate to the active site. The active site of SV-LAAO comprises FAD and the amino acid residues Arg90, His223, Phe227, Lys326, Tyr372, and Trp375 and a conserved water molecule near FAD and Lys326 (**Figure 2D**). The FAD, Lys326, and the conserved water molecule form a triad Lys326-Water-N5 (FAD) upon substrate binding. The His233 deprotonates the α-amino group of the substrate (amino acid) during the deamination reaction.

### Ligand/Substrate-Binding Sites

**Figure 4:**
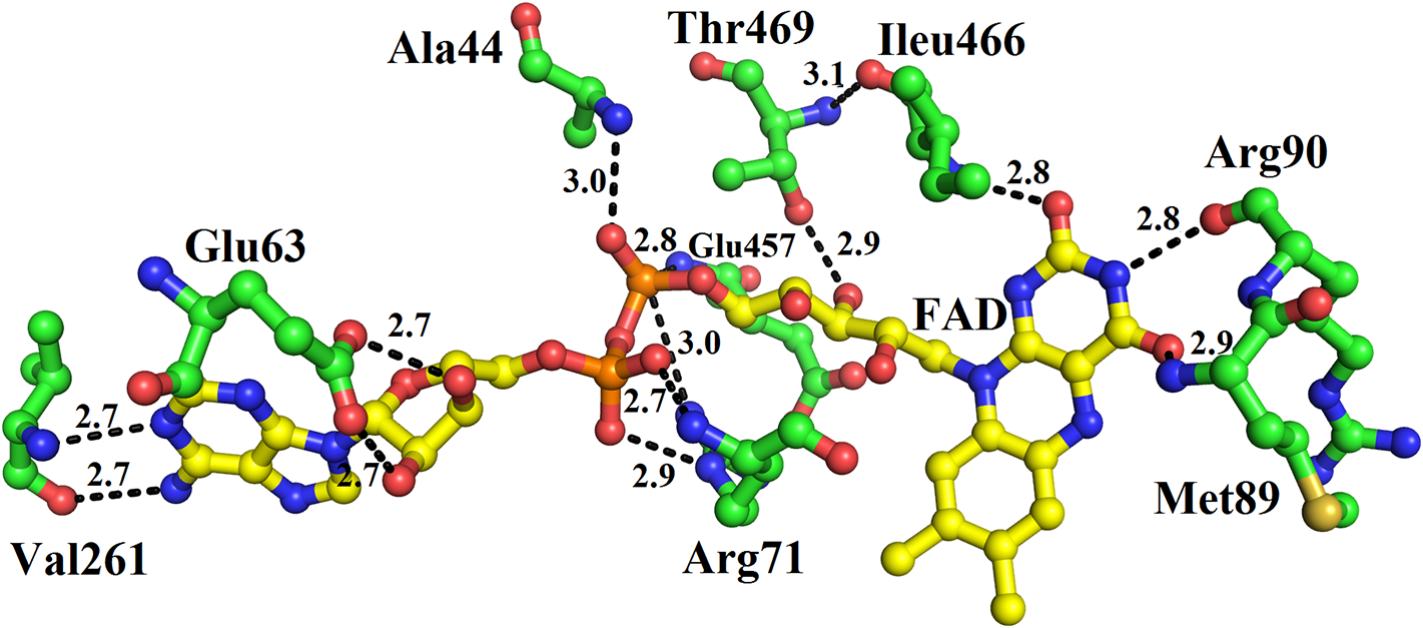
FAD-binding amino acid residues of SV-LAAO. The FAD is shown as yellow sticks and the amino acid residues as green sticks.

The FAD is located in between the cofactor and substrate binding domains and is buried deep in the protein. The FAD makes intensive contacts with the amino acid residues from both of these domains and several water molecules. These amino acid residues include Ala44, Glu63, Arg71, Met89, Arg90, Val261, Ileu466, and Thr469 (**Figure 4**). The flavin or Isoalloxazine ring makes contact with Met89, Arg90 and Ileu466. The adenine moiety of FAD is bonded to Glu63 and Val261 while the phosphate and sugar part is bonded to Arg71, Ala44, Glu467, and Thr469 (**Figure 4**).

**Figure 5:**
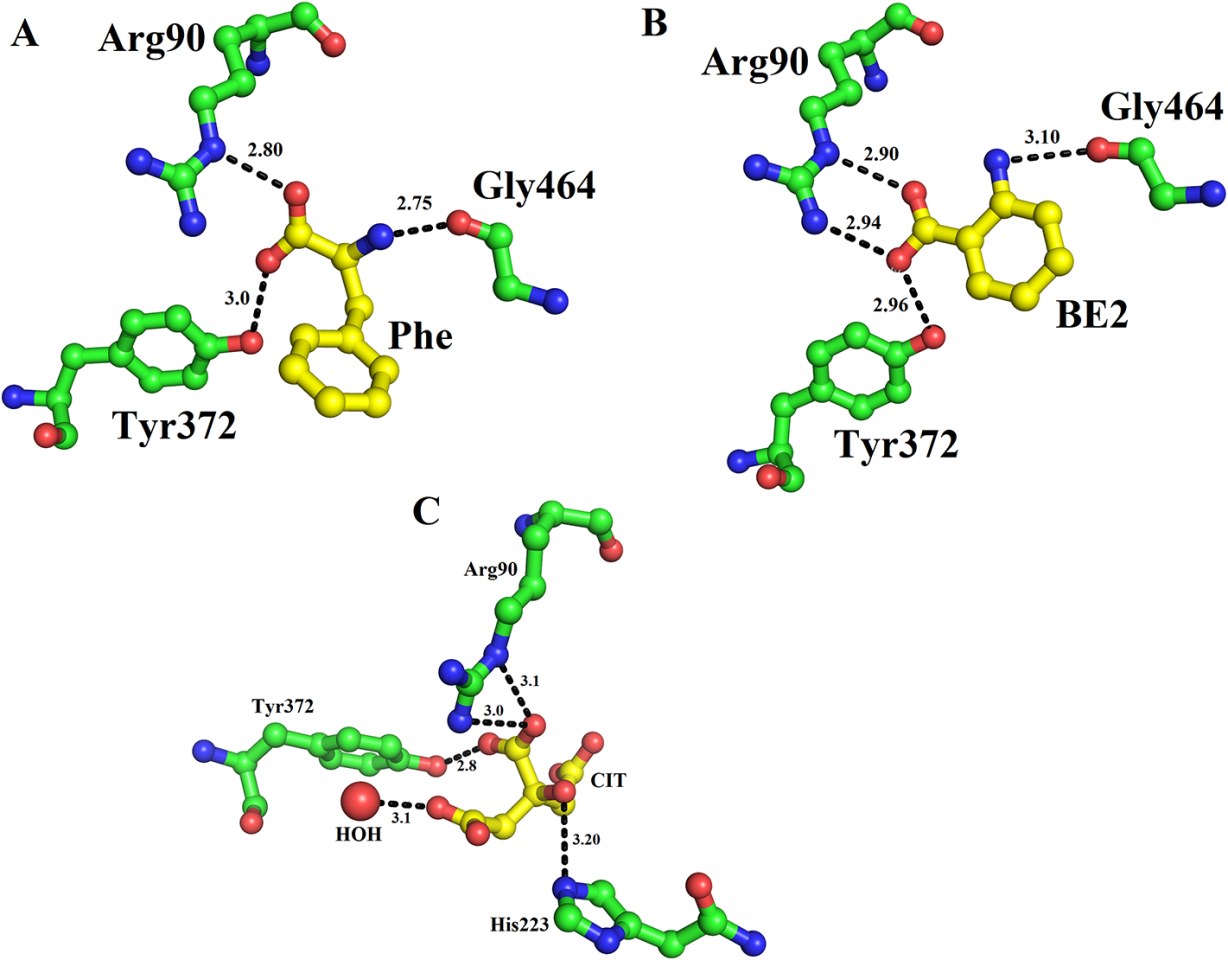
Substrate/ligand bind amino acid residues of SV-LAAOs. Structure of *Calloselasma rhodostoma* with bound **(A)** L-Phenylalanine **(B)** BE2- 2-aminobenzoic acid **(C)** Citrate. The substrate/ligands are shown as yellow sticks and the amino acid residues as green sticks.

The structure of LAAO from *C. rhodostoma* determined with the bound citrate, 2-amino benzoic acid and L-phenylalanine provides insights into the inhibitors/substrate binding (**Figures 5A–C**). In all three cases the amino acid residues involved are Arg90, Tyr372 and Gly464 (**Figures 5A–C**). Sequence alignment analysis indicates that the ligand/substrate binding amino acid residues are fully conserved among the SV-LAAOs (**Figure 1**).

### Zinc Binding Sites

Zinc ions have been found in the crystal structure of LAAOs from *Vipera ammodytes ammodytes* and *B. atrox* (Georgieva et al., 2011; Feliciano et al., 2017). Both enzymes were crystallized in the presence of zinc (zinc acetate and zinc sulfate) (Georgieva et al., 2011; Feliciano et al., 2017). However, LAAOs from *B. jararacussu* and *C. rhodostoma* have no zinc ion in the solution or crystal form (Ullah et al., 2012a; Moustafa et al., 2006), which indicates that the LAAOs from *V. ammodytes ammodytes* and *B. atrox* may have taken the zinc ions from the crystallization solution (Feliciano et al., 2017).

**Figure 6:**
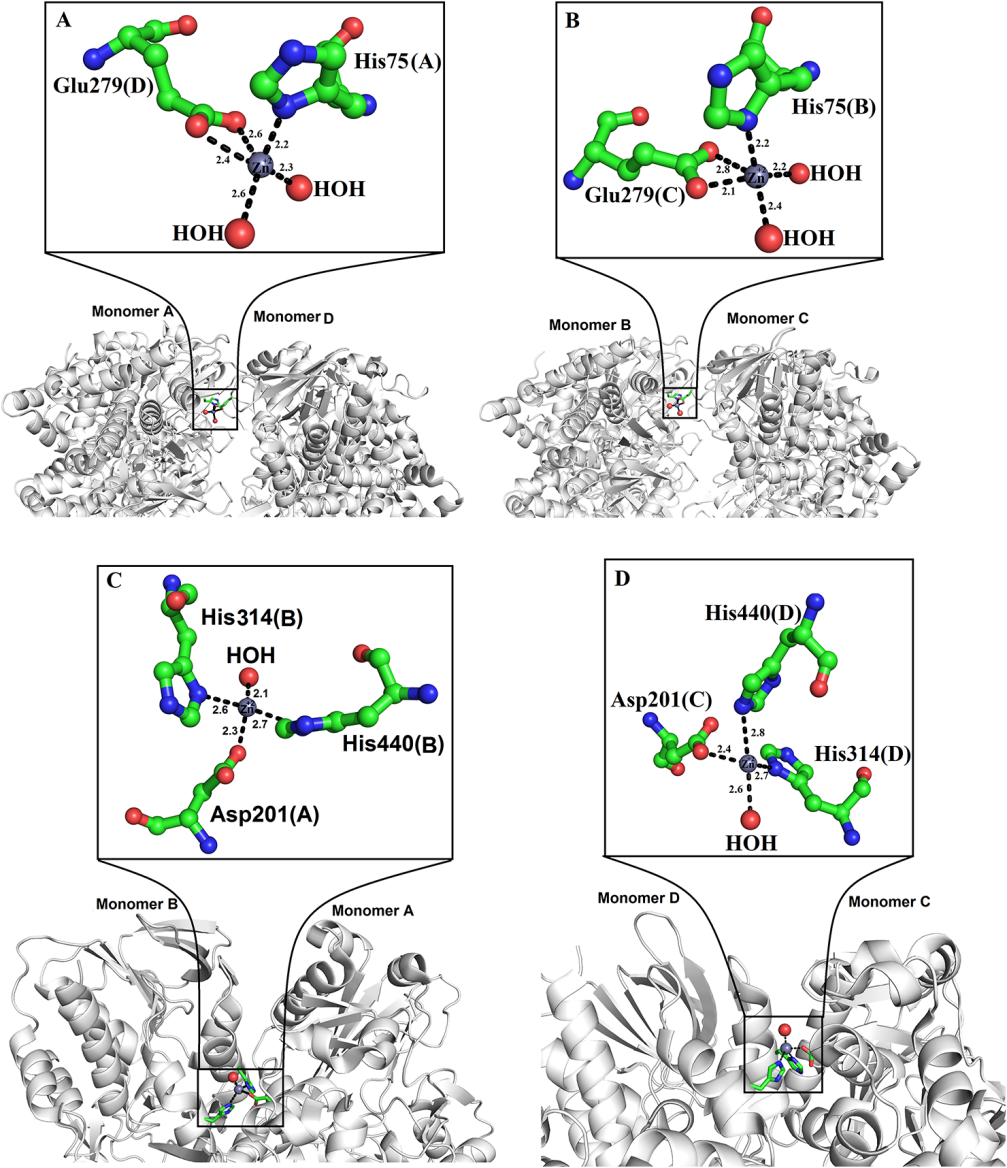
Zinc binding sites of SV-LAAOs **(A**, **B)** Zinc binding site of L-amino acid oxidase from *Vipera ammodytes ammodytes*, (monomers A and D and B and C) **(C**, **D)** Zinc binding site of L-amino acid oxidase from *Bothrops atrox* (monomers A and B and C and D). The zinc ions and water molecules are shown as gray and red spheres respectively and the amino acid residues as green sticks.

In the tetrameric structure of *V. ammodytes ammodytes* LAAO, the four zinc ions are tetrahedrally coordinated. The zinc ions that connect the monomer A to monomer D are coordinated by His75, Glu279 and two water molecules (**Figure 6A**), while the other zinc ion connecting monomer B and C is also coordinated by His75, Glu279 and two water molecules (**Figure 6B**). The crystal structure of *B. atrox* contains eight zinc ions in which the two zinc ions that connect monomers A and B and monomers C and D are correctly coordinated (**Figures 6C, D**). The remaining six zinc ions are located at the surface of the protein and they are poorly coordinated as confirmed by CheckMyMetal (CMM) (Zheng et al., 2014). In the LAAOs from *V. ammodytes ammodytes* and *B. atrox* zinc ions have been found to stabilize the dimers and these are considered important for the biological activities of these enzymes (Georgieva, et al., 2008; Feliciano et al., 2017). The inhibition and activation by metal ions have been investigated for LAAOs from *Crotalus adamanteus*, *Lachesis muta, Bothrops Brazili,* and *Agkistrodon blomhoffii ussurensis* (Bender and Brubacher, 1977; Cisneros, 1996; Solis et al., 1999; Sun et al., 2010). The enzymatic activity of *C. adamanteus* LAAO is enhanced by Mg^+2^ and that of *L. muta* and *B. brazili* LAAOs is inhibited by zinc ion. The zinc ion does not affect the enzymatic activity of *A. blomhoffii ussurensis* LAAO; however, it is important for the structural integrity of the protein (Sun et al., 2010).

### Glycosylation

**Figure 7:**
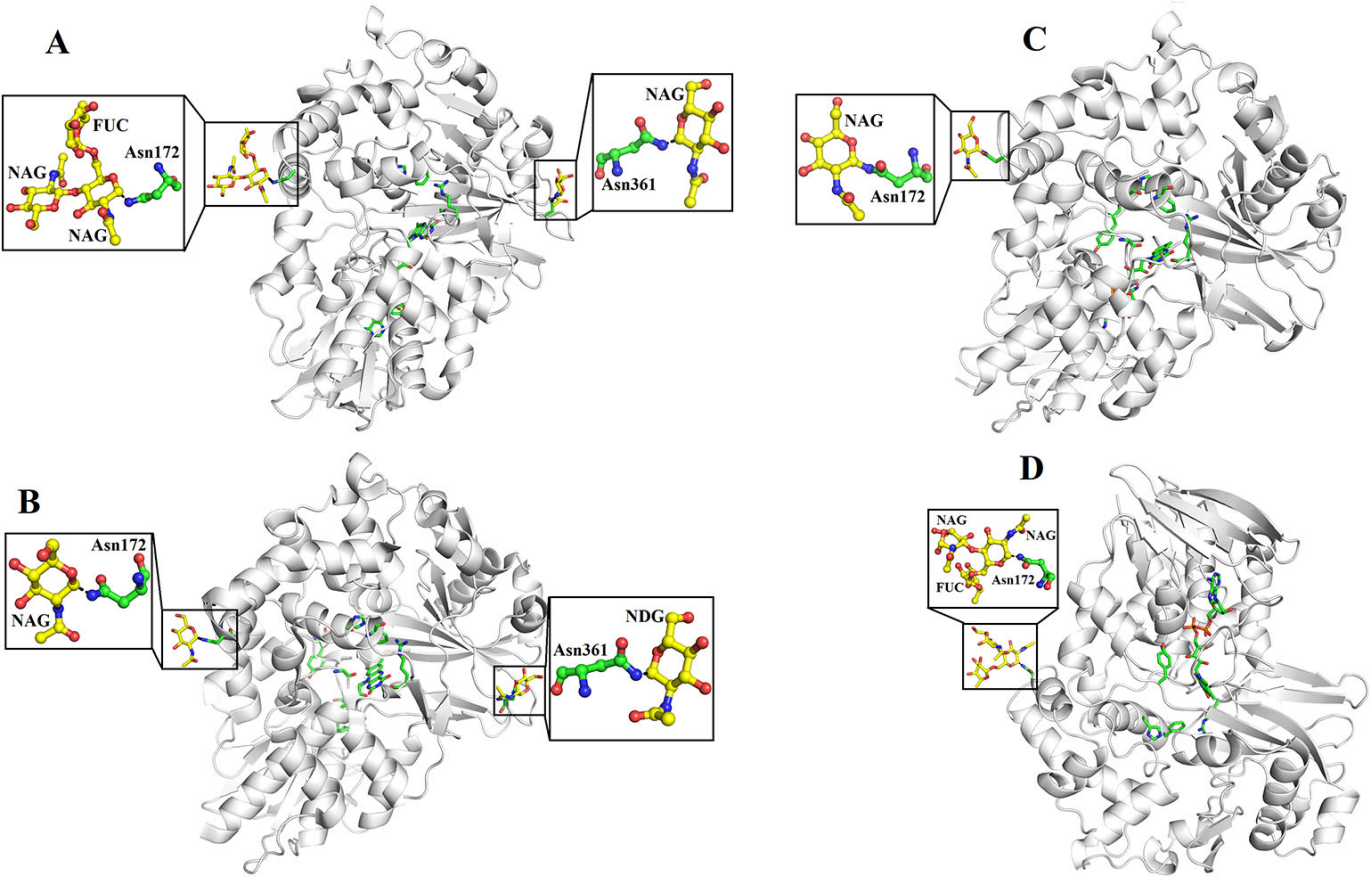
Glycosylation sites of **(A)** L-amino acid oxidase from *Calloselasma rhodostoma* **(B)** L-amino acid oxidase from *Agkistrodon halys pallas* **(C)** Structure of native L-amino acid oxidase from *Vipera ammodytes ammodytes* **(D)** Crystal structure of L-amino acid oxidase from *Bothrops atrox*. The carbohydrate moiety (NAG, FUC) is shown as yellow sticks and the Asn (Asn172 and Asn361) are shown as green sticks.

SV-LAAOs are glycosylated proteins with 3–4% carbohydrate moiety (deKok and Rawitch, 1969; Hayes and Wellner, 1969). In some cases, the carbohydrate contents may reach up to 12% of the total molecular mass of the protein (Alves et al., 2008). The LAAOs from *C. rhodostoma* and *Agkistrodon halys pallas* contain two glycosylation sites (Asn172 and Asn361) (**Figures 7A, B**) and that of *V. ammodytes ammodytes* and *B. atrox* have a single glycosylation site (Asn172) (**Figures 7C, D**). The glycosylation sites are fully conserved in SV-LAAOs (**Figure 1**). In the case of LAAOs from *C. rhodostoma* and *B. atrox* Asn172 has three carbohydrate moieties, NAG-FUC-NAG (NAG: N-Acetyl-D-Glucosamine; FUC: alfa-L-Fucose) (**Figures 7A, D**), while Asn361 has one NAG in the former and the latter lacks a carbohydrate moiety at this position. The LAAOs from *A. halys pallas* has a single carbohydrate moiety at both positions 172 and 361 (**Figure 7B**). The *V. ammodytes ammodytes* LAAO has a single glycosylation site (Asn172) and only one NAG molecule (**Figure 7D**). The *B. jararacussu* LAAO lacks carbohydrates at both positions 172 and 361 however; biochemical study has shown that *B. jararacussu* LAAO contains carbohydrates (França et al., 2007; Carone et al., 2017). The LAAOs from *Daboia russelii* and *Trimeresurus stejnegeri* venom have been shown to contain three glycosylation sites at Asn172, 194, and 361 (Zhang et al., 2003; Chen et al., 2012). The glycan moiety in SV-LAAOs is bis-sialylated, biantennary, and core-fucosylated dodecasaccharides (Geyer et al., 2001). The glycan moiety, particularly at the position 172 lies near to the O_2_ entrance and H_2_O_2_ exit tunnel and have been implicated to increases the concentration of the later upon attachment to the cell surface (Suhr and Kim, 1996; Torii et al., 1997; Ande et al., 2006). Thus the glycan moiety plays an important role in the attachment of LAAO to the cell surface thereby increasing the concentration of H_2_O_2_ which leads to the apoptosis (Ande et al., 2006). Evidence for the direct attachment of SV-LAAOs to mouse lymphocytic leukemia and endothelial KN-3 cells (Suhr and Kim, 1996), human umbilical vein endothelial cell and promyelocytic leukemia HL-60, and human ovarian carcinoma A2789 cells have been confirmed using Fluorescence microscopy with a fluorescence label LAAO (Suhr and Kim, 1996). The removal of glycan moiety from the SV-LAAOs drastically decreases the apoptotic activity of these enzymes; however, it does not affect their catalytic activity (Geyer et al., 2001; Stabeli et al., 2004; Izidoro et al., 2006; Chen et al., 2012).

### Structural Comparison Among SV-LAAOs

**Figure 8:**
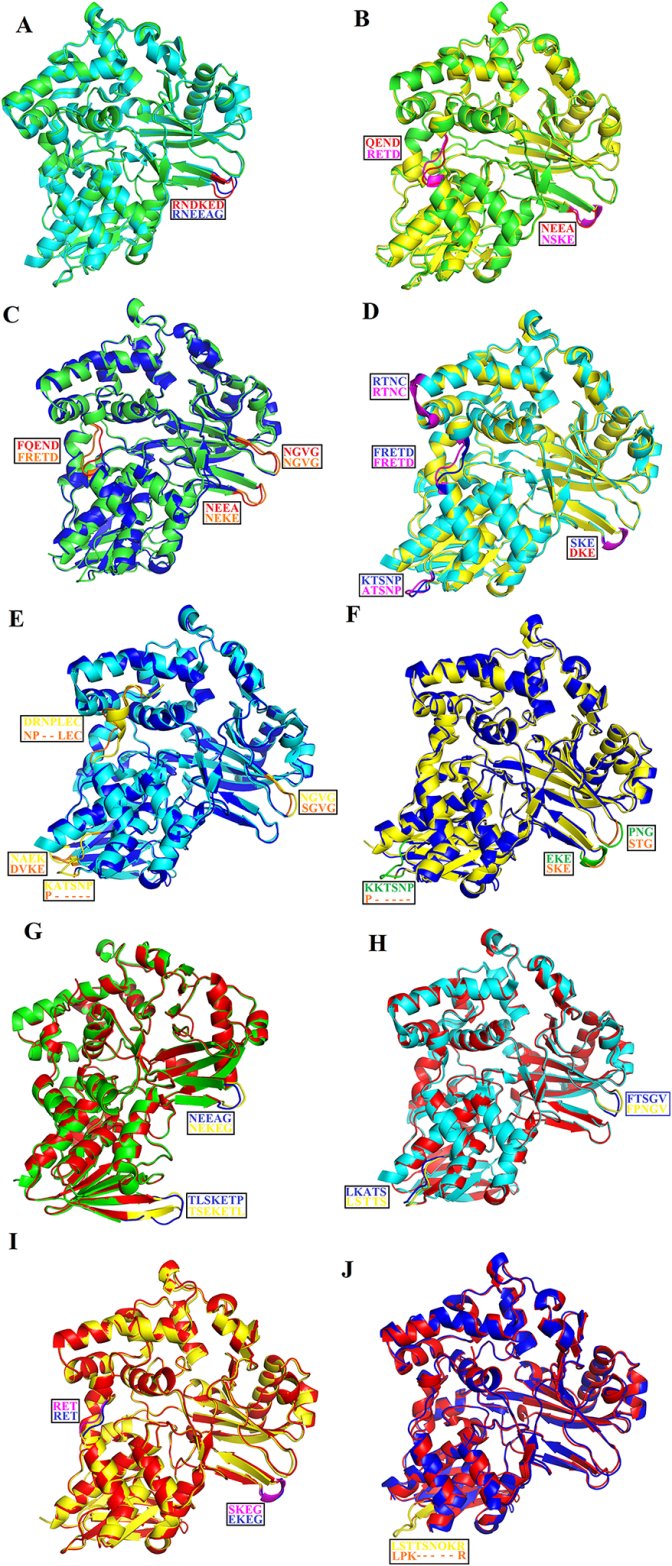
Structural alignment among SV-LAAOs **(A)** *Calloselasma rhodostoma* LAAO (green) align with *Agkistrodon halys pallas* LAAO (cyan) **(B)** *C. rhodostoma* LAAO (green) align with *Vipera ammodytes ammodytes* LAAO (yellow) **(C)** *C. rhodostoma* LAAO (green) align with *Bothrops jararacussu* LAAO (blue) **(D)** *A. halys pallas* LAAO (cyan) align with *V. ammodytes ammodytes* LAAO (yellow) **(E)** *A. halys pallas* LAAO (cyan) align with *B. jararacussu* LAAO (blue) **(F)** *V. ammodytes ammodytes* LAAO (yellow) align with *B. jararacussu* LAAO (blue) **(G)** *C. rhodostoma* LAAO (green) align with *Bothrops atrox* LAAO (red) **(H)** *A. halys pallas* LAAO (cyan) align with *B. atrox* LAAO (red) **(I)** *V. ammodytes ammodytes* LAAO (yellow) align with *B. atrox* LAAO (red) **(J)** *B. jararacussu* LAAO (blue) align with *B.* *atrox* LAAO (red). The loops showing variable amino acid residues and length are highlighted. The amino acid residues showing differences are shown in the box.

The overall three-dimensional structures of SV-LAAOs align well to each other (**Figures 8A–J**). They have the same three-dimensional structural folds that contain three domains namely FAD-binding domain, substrate-binding domain and a helical domain. The Root Mean Square Deviation (RMSD) value for the structural alignment among SV-LAAOs range from 0.30–0.66 Å, with an average RMSD value of 0.46 Å (**Table 4**). The main differences are found in the loop regions (**Figures 8A–J**). The amino acid sequence and length of the loops vary in these regions. This may be important in variable substrate specificity.

**Table 4:** Root mean square deviation of SV-LAAOs structural alignment.

Protein	RMSD (Å)
1F8R align 1REO	0.30
1F8R align 3KVE	0.51
1F8R align 4E0V	0.61
1F8R align 5TS5	0.36
1REO align 3KVE	0.49
1REO align 4E0V	0.50
1REO align 5TS5	0.34
3KVE align 4E0V	0.66
3KVE align 5TS5	0.36
4E0V align 5TS5	0.51

The alignment of the two structures was carried out using the PyMOL Molecular graphic visualization program and the all-atom RMSD values were calculated using the same program.

**Figure 9:**
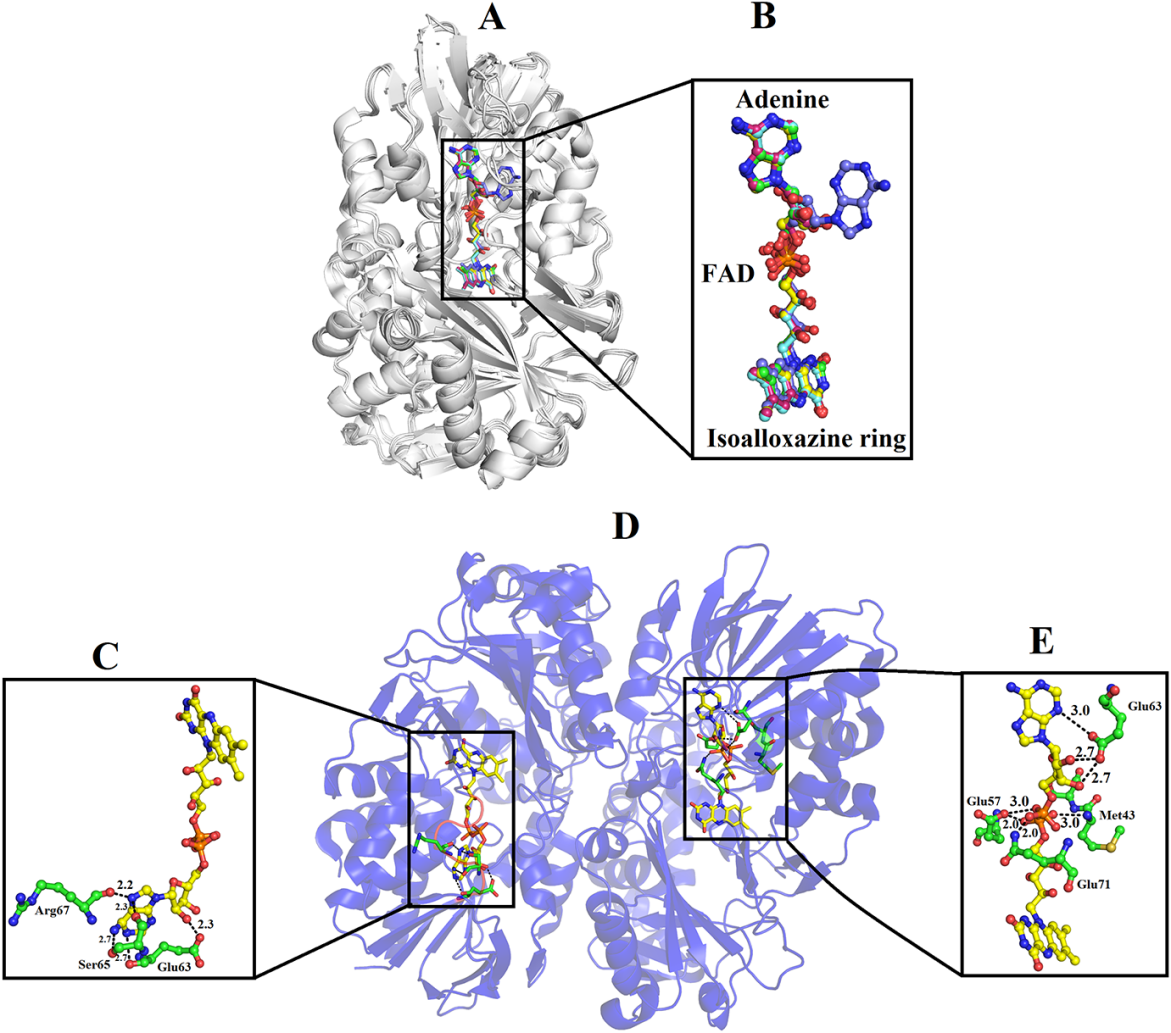
Binding mode of FAD in SV-LAAOs **(A)** structural alignment among SV-LAAOs **(B)** FAD-binding in SV-LAAO **(D)** Two ways of FAD-binding in *Bothrops jararacussu* LAAO **(C)** FAD-binding in monomer A **(E)** FAD-binding in monomer B. The FAD has been shown as yellow sticks and the amino acid residues as green sticks.

In the crystal structure of all SV-LAAOs the adenosyl group of FAD has a normal canonical form in which it is stabilized by Van der Waals contacts and the ribose and di-phosphoryl groups that are tightly bonded to E63, Q71 and E457 side chains and backbone N atoms from M43 and S44 (**Figures 9A, B, D, E**). However, in the crystal structure of *B. jararacussu* LAAO the adenosyl group was found in different conformation from the normal canonical binding mode (**Figures 9B, C**). In this novel conformation, the adenosyl group of the FAD flips towards loop 62–71 and is stabilized by the interaction with amino acid residues (E63, S65 and R67, and a large number of hydrophobic contacts) from this loop (**Figure 9C**). Due to this new conformation, the active site cleft volume of *B. jararacussu* LAAO increases which further modifies the solvent accessibility to the FAD-binding domain that was previously occupied by the adenosyl group in the canonical-binding mode. This may also contribute to the variable substrate specificity of this enzyme (Ullah et al., 2012b).

### Variable Substrate Specificity Among SV-LAAOs

The substrate (amino acid) recognition amino acid residues comprising Arg90, Arg322, Tyr372, Ileu430, and Trp465 are fully conserved among all SV-LAAOs (**Figure 1**). However, LAAOs from various snake species displayed variable substrate specificity (Moustafa et al., 2006; Ullah et al., 2012b; Bregge-Silva et al., 2012; Chen et al., 2012). For example LAAOs from *B. jararacussu*, *L. muta* and *R. viper* display preference for hydrophobic amino acid (L-Met, L-Leu, L-Phe, L-Ileu, L-Trp, and L-Tyr) with large side chain (Bregge-Silva et al., 2012; Chen et al., 2012; Ullah et al., 2012b) while the LAAO from *C. rhodostoma* shows broad specificity toward their substrate (Moustafa et al., 2006). Interestingly the *O. hannah* LAAO has shown a high substrate preference for L-Lysin (Tan and Saifuddin, 1991). The narrow and broad specificity of SV-LAAOs can be explained based on the amino acid residues difference in the loop regions, active site cavity volume and its average depth and surface charge distribution.

**Figure 10:**
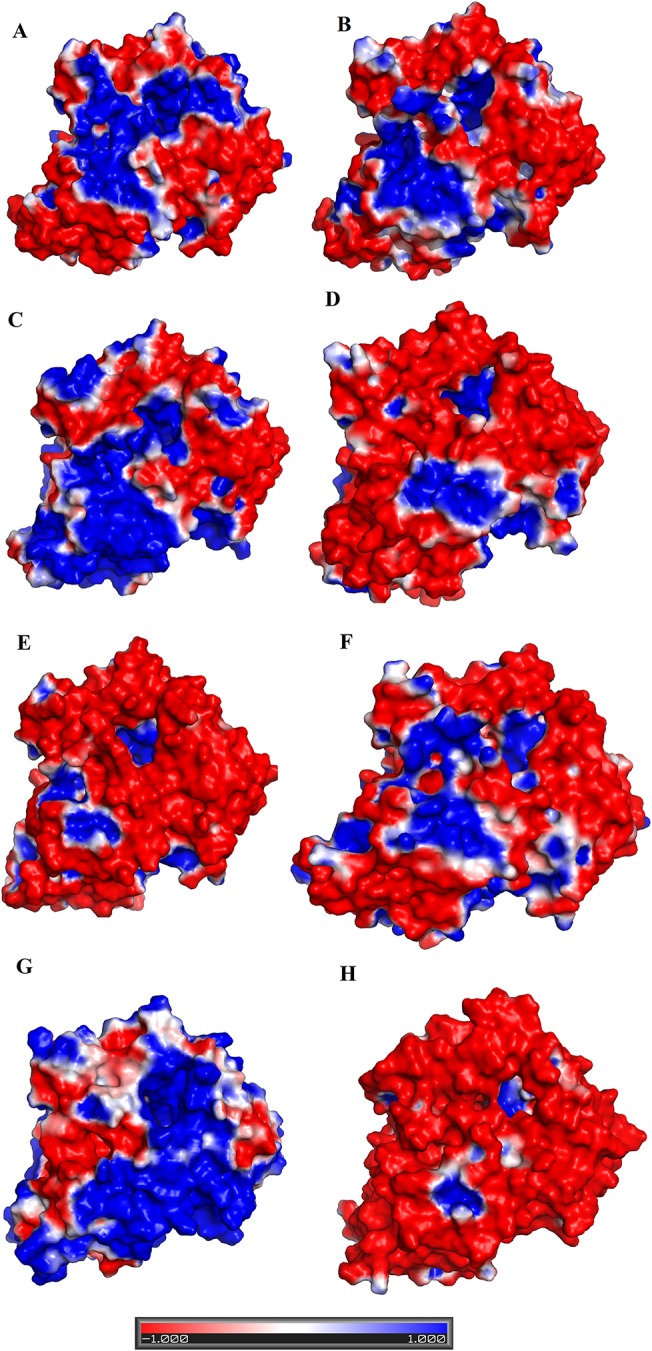
Surface charge distribution of SV-LAAOs **(A)** *Calloselasma rhodostoma* LAAO **(B)** *Agkistrodon halys pallas* LAAO **(C)** *Vipera ammodytes ammodytes* LAAO **(D)** *Bothrops jararacussu* LAAO **(E)** *Bothrops atrox* LAAO **(F)** *Lachesis muta* LAAO (homology model) **(G)** *R. viper* LAAO (homology model) **(H)** *Ophiophagus hannah* LAAO (homology model). The red, blue and white colors represent negative, positive and neutral charges respectively.

The analysis of structural alignment among SV-LAAOs from various snake species shows some differences in their three-dimensional structure that is confined to the loop regions (**Figures 8A–J**). Due to these differences, the surface charge distribution varies in these enzymes (**Figures 10A–E**). The surface charge distribution analysis indicates that LAAOs having broad specificity have their surfaces partially negative and partially positive (**Figures 10A–C**). While others having specificity for hydrophobic amino acids have highly negatively charged surface around the active site cleft (**Figures 10D–F**).

The active site cavity volume and average depth also vary in these enzymes (**Table 5**). It has been shown that the SV-LAAOs with broad substrate specificity (LAAO from *C. rhodostoma*) have small active site cavity volume (4719.94 Å^3^) and average depth (16.55 Å) (**Table 5**). However, the others SV-LAAOs with narrow substrate specificity have large active site cavity volume (8,469.14–13,670.44 Å^3^) and average depth (18.23–23.58 Å) (**Table 5**). The unique preference of *O. hannah* LAAO toward L-lysine (basic amino acid, with a positive charge) as a substrate can also be explained based on the surface charge distribution (**Figure 10H**) and active site cavity volume and its average depth (**Table 5**). The overall surface charge of this enzyme is highly negatively charged which attract this amino acid (**Figure 10H**). However, in the case of other SV-LAAOs, the overall surface charge is partially negative and partially positive (**Figures 10A–G**). The average active site cavity volume and depth is also very small for O*. hannah* LAAO when compared to the other SV-LAAOs (**Table 5**).

**Table 5:** Average active site cavity volume and average active site cavity depth of SV-LAAOs and their mammalian and bacterial counterpart.

Protein	Average volume (Å^3^)	Average depth (Å)
1F8R	4719.94	16.55
1REO	10710.98	21.84
3KVE	7425.42	23.58
4E0V	8469.14	18.23
5TS5	12895.45	21.67
RV-LAAO- homology model	13670.44	21.85
LM-LAAO- homology model	9656.30	21.91
*O. hannah*- homology model	2688.61	8.54

1F8R: Calloselasma rhodostoma LAAO, 1REO: Agkistrodon halys pallas LAAO, 3KVE: Vipera ammodytes ammodytes LAAO, 4E0V: Bothrops jararacussu LAAO, 5TS5: Bothrops atrox LAAO, R. viper LAAO (homology model), Lachesis muta LAAO (homology model), Ophiophagus Hannah LAAO (homology model). The average active site cavity volume and average active site cavity depth of the proteins were calculated using the Pdbsum online server (Laskowski et al., 2018).

### Catalytic Mechanism

The active site of SV-LAAO is located deeply within the enzyme with a long funnel-like entrance (25 Å). The walls of the funnel are lined with the hydrophilic and hydrophobic amino acid residues that direct the substrate to the active site (Pawelek et al., 2000; Moustafa et al., 2006). The active site comprises of the cofactor FAD and amino acid residues Arg90, His223, Phe227, Lys324, Tyr372, Ileu374, Ileu430, and Trp465 (**Figures 1**, **2D**, and **4**) (Georgieva et al., 2011; Pawelek et al., 2000; Moustafa et al., 2006).

The cofactor FAD (substrate-bound and reduced state) acts as a receptor of the hydride from the substrate C-alpha atom to the N5 atom of the flavin isoalloxazine ring system (Moustafa et al., 2006). The Arg90 interact with the carboxylic acid group of the amino acid substrate and keeps it in the specific orientation for the catalysis (Georgieva et al., 2010). The amino acid residues Phe227, Tyr372 and Trp465 stabilize the isoalloxazine part of the FAD cofactor. The Ile374 and Ile430 constitute the hydrophobic substrate-binding site and preferably bind the amino acids (substrate) with non-polar side chains. A conserved water molecule near Lys322 and FAD cofactor (N5 atom of the flavin isoalloxazine ring) has been encountered in the crystal structure of *C. rhodostoma* LAAO with bound L-phenylalanine (Moustafa et al., 2006) and also in the native structure of *V. ammodytes ammodytes* (Georgieva, et al., 2010). This water molecule makes a triad Lys322-Water- N5 of FAD (Isoalloxazine ring), only upon substrate binding (Moustafa et al., 2006; Georgieva, et al., 2010). This water molecule is important for FAD reduction and the formation of H_2_O_2_ (Moustafa et al., 2006).

**Figure 11:**
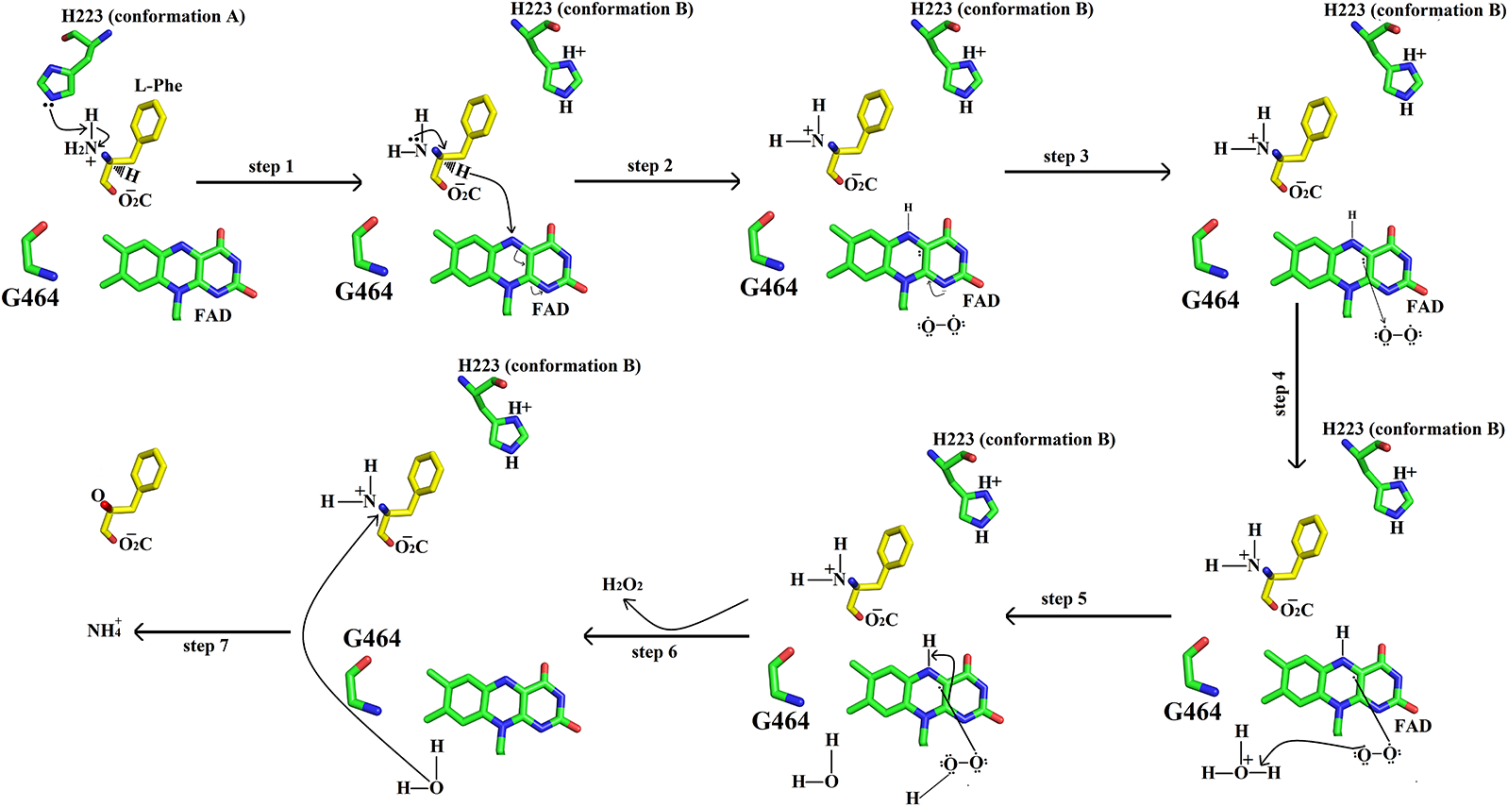
Catalytic mechanism of SV-LAAO. The amino acid residues (His223, Gly464) and FAD are displayed as green sticks. The substrate (L-phenylalanine) is shown as yellow sticks. Only isoalloxazine ring of FAD has been shown here.

The catalytic mechanism involves two reactions namely reductive half-reaction and oxidative half-reaction. The protonated amino acid (substrate) in the zwitterionic form enters the active site of the enzyme through the funnel-shaped channel (Pawelek et al., 2000). In the funnel, His233 and Arg322 block the substrate as they change their conformation due to the zwitterionic form of the substrate (**Figure 11**) (Moustafa et al., 2006). The His233 then removes a proton from the α-amino group of the substrate. After deprotonation, the substrate is further modified by transferring the electrons from the α-nitrogen to the α-carbon atom of the substrate. This makes the substrate more active and thus it transfers a hydride ion to the N5 of the FAD cofactor and reduces it. The substrate changes to the imino form during this step. During the oxidative half-reaction, the FAD is oxidized by O_2_. The O_2_ takes one electron from the FAD π-electrons and becomes attached to the other electron. In the same time, the O_2_ also abstracts a hydrogen ion from the water molecule (hydronium ion) and hydrogen from the FAD which it has taken previously from His223. In this way, an H-O-O-H (H_2_O_2_) is formed. The bond between the H_2_O_2_ and FAD breaks and the H_2_O_2_ is released. The water molecule near Lys322 provides oxygen to the imino acid changing it to keto acid and the hydrogen to the NH_2_ converting it to NH_3_.

### Inhibition of SV-LAAO

**Figure 12:**
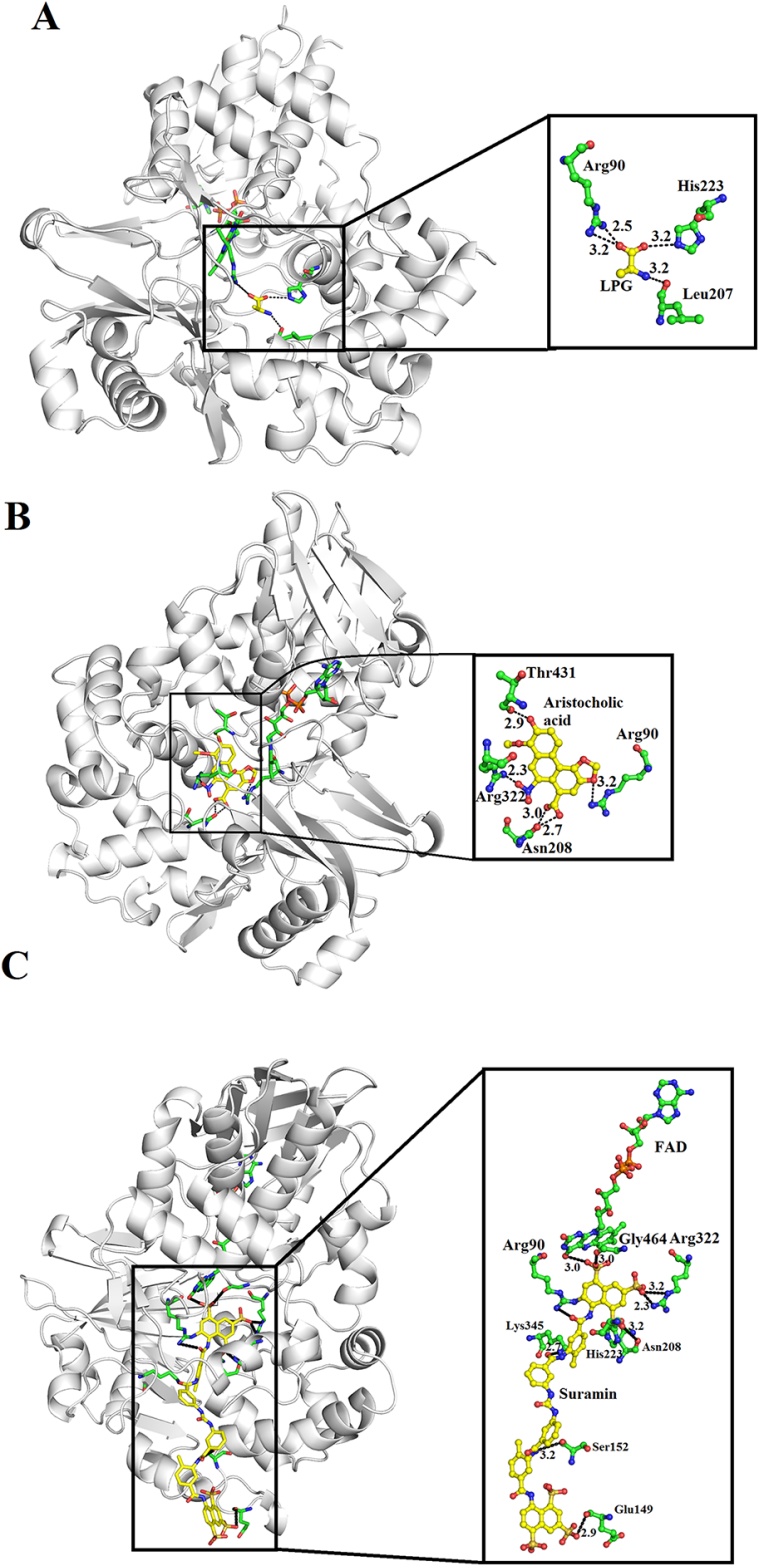
Inhibition of SV-LAAOs by L-propargylglycin, Aristocholic acid and suramin **(A)** *Vipera ammodytes ammodytes* LAAO with bound L-propargylglycin **(B)** *V. ammodytes ammodytes* LAAO with bound Aristocholic acid **(C)** *V. ammodytes ammodytes* LAAO with bound suramin. The amino acid residues interacting with the inhibitors are shown as green sticks. The inhibitors are shown as yellow sticks.

SV-LAAOs are inhibited by L-propargylglycin, Aristocholic acid and suramin (Mitra and Bhattacharyya, 2013; Bhattacharjee et al., 2017). The L-propargylglycin reversibly inhibits the *C. adamanteus* and *C. atrox* LAAOs by covalent modification (Mitra and Bhattacharyya, 2013). This inhibitor binds to amino acid residues Arg90, His233 and Leu207 (**Figure 12A**). The Arg90 and His233 are important for SV-LAAO activity as inhibiting these two leads to the inhibition of the enzyme. The Aristocholic acid and its derivatives bind to the amino acid residues Arg90, Asn208, Arg322, and Thr431 (**Figure 12B**), while suramin binds to Arg90, Glu149, Ser152, His223, Asn208, Lys345, and Arg322, Gly464, and FAD (**Figure 12C**). The Aristocholic acid and its derivatives and suramin inhibit the function of these enzymes by binding to the key amino acid residues (Arg90, His223, and Arg322).

## Interaction of SV-LAAO With Membrane

**Figure 13:**
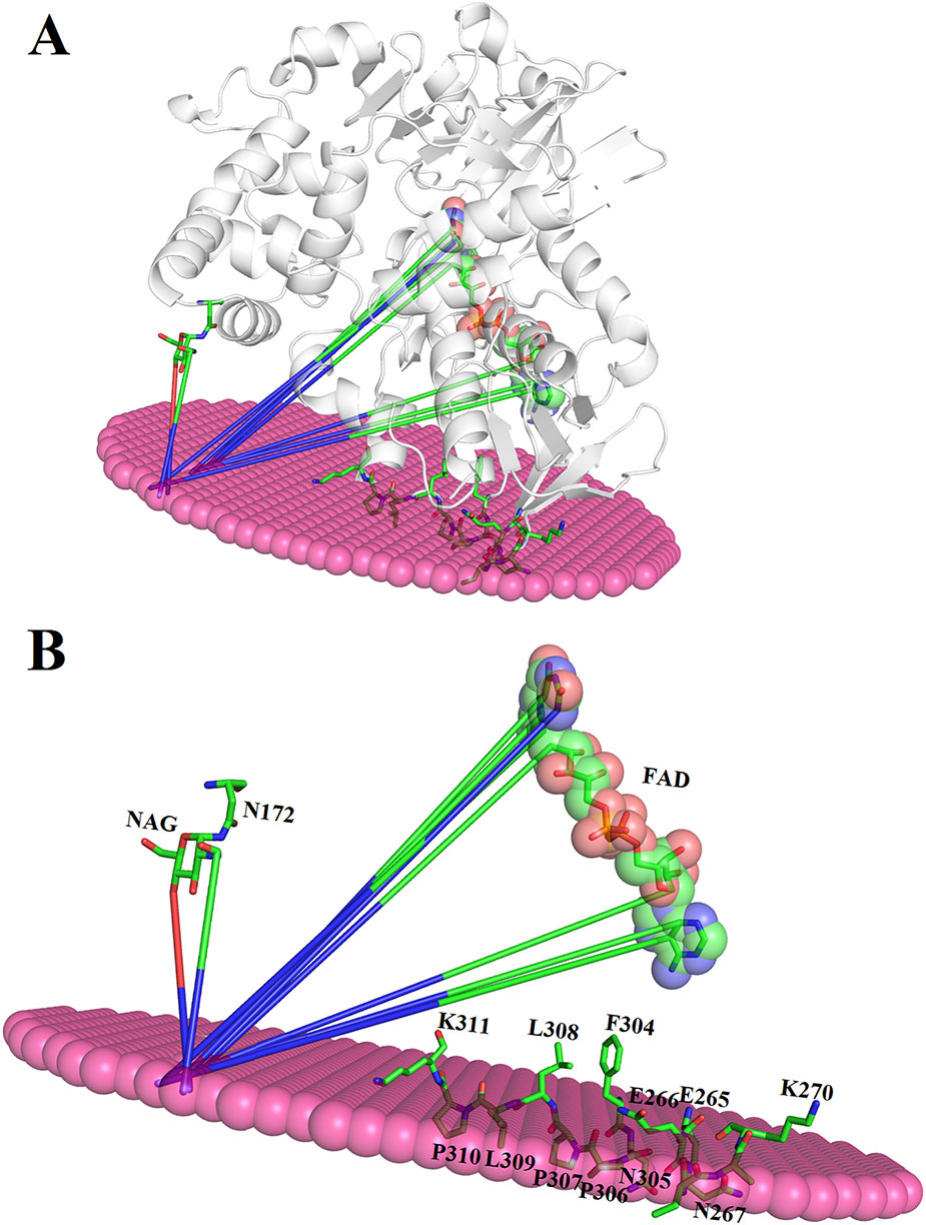
**** **(A)** Interaction of SV-LAAO with membrane. The binding was predicted using PPM server **(B)** Parts (amino acid residues, FAD and NAG) of the SV-LAAO interacting with the membrane.

The binding between membrane and SV-LAAO was predicted using PPM server (Lomize et al., 2012). The SV-LAAO membrane interaction is mediated by the contacts from FAD, glycan moiety from Asn172 and the amino acid residues from the loops (**Figure 13A**). The amino acid residues that make contact with membrane include Glu265, Glu266, Asn267, Asn305, Pro306, Pro307, Leu309, and Pro310 (**Figure 13B**). The interaction of SV-LAAO with membrane increases the concentration of H_2_O_2_ that is considered important for the apoptotic activity of this enzyme (Suhr and Kim, 1996; Torii et al., 1997; Ande et al., 2006).

## Conclusion and Future Directions

SV-LAAO is the most potent apoptotic agents in snake venom. The earliest study of these enzymes was concerned with their enzymatic properties and industrial applications; however, recently more attention has been given to their structure-functional relationship, mechanism of action, therapeutic potentials and biotechnological applications. Sequence analysis indicates a high sequence identity among SV-LAAOs and low identity with the bacterial, fungal, plants and mammalian homologs. Their three-dimensional structure has three well-defined domains namely a FAD-binding domain, a substrate-binding domain and a helical domain. The helical domain makes a funnel-like structure that directs the substrate to the active site of these enzymes. The sequence and structural analysis indicate some differences in amino acid residues in the loop regions. These differences change the surface charge distribution, average active site cavity volume and depth which may impart variable substrate specificity to these enzymes. The inhibition of these enzymes by synthetic inhibitors (L-propargylglycin, Aristocholic acid and its derivatives and suramin) can lead to better treatment of snakebite envenomation. Further investigations are necessary to use these enzymes as a therapeutic agent in cancer and HIV-AIDS treatment.

## Author Contributions

AU has designed the project, written, drafted, and reviewed the current manuscript.

## Conflict of Interest

The author declares that the research was conducted in the absence of any commercial or financial relationships that could be construed as a potential conflict of interest.
