# Supplementary material for: Structure–Function Studies and Mechanism of Action of Snake Venom L-Amino Acid Oxidases
Source: Front Pharmacol. 2020 Feb 25;11:110. doi: 10.3389/fphar.2020.00110 (PMC7052187; doi:10.3389/fphar.2020.00110)
Supplement: Supplementary file 1 [file DataSheet_1.docx]

**Supplementary materials**


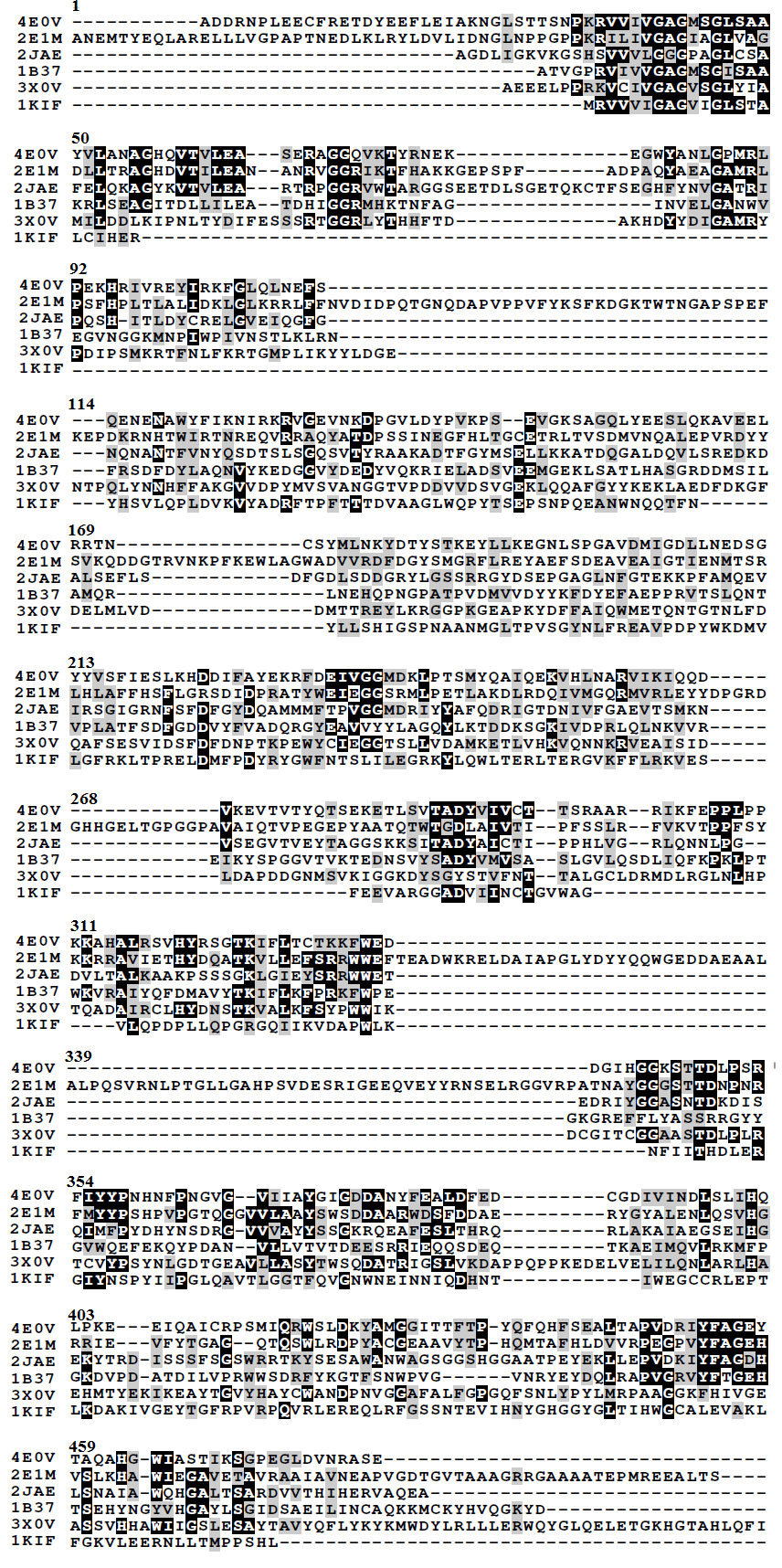


#### Figure S1: Sequences alignment among snake venom L-amino acid oxidase, bacterial (L-Glutamate Oxidase), *Zea mays* (polyamine oxidase), Fungi (L-lysine oxidase) and pig kidney (D-amino acid oxidase). 4E0V: *Bothrops jararacussu* LAAO, 2E1M: Streptomyces sp. L-Glutamate Oxidase, 2JAE: *Rhodococcus opacus* L-amino acid oxidase, 1B37: *Zea mays* polyamine oxidase, 3X0V: *Hypocrea rufa* L-lysine oxidase, 1KIF: Pig kidney D-amino acid oxidase

**
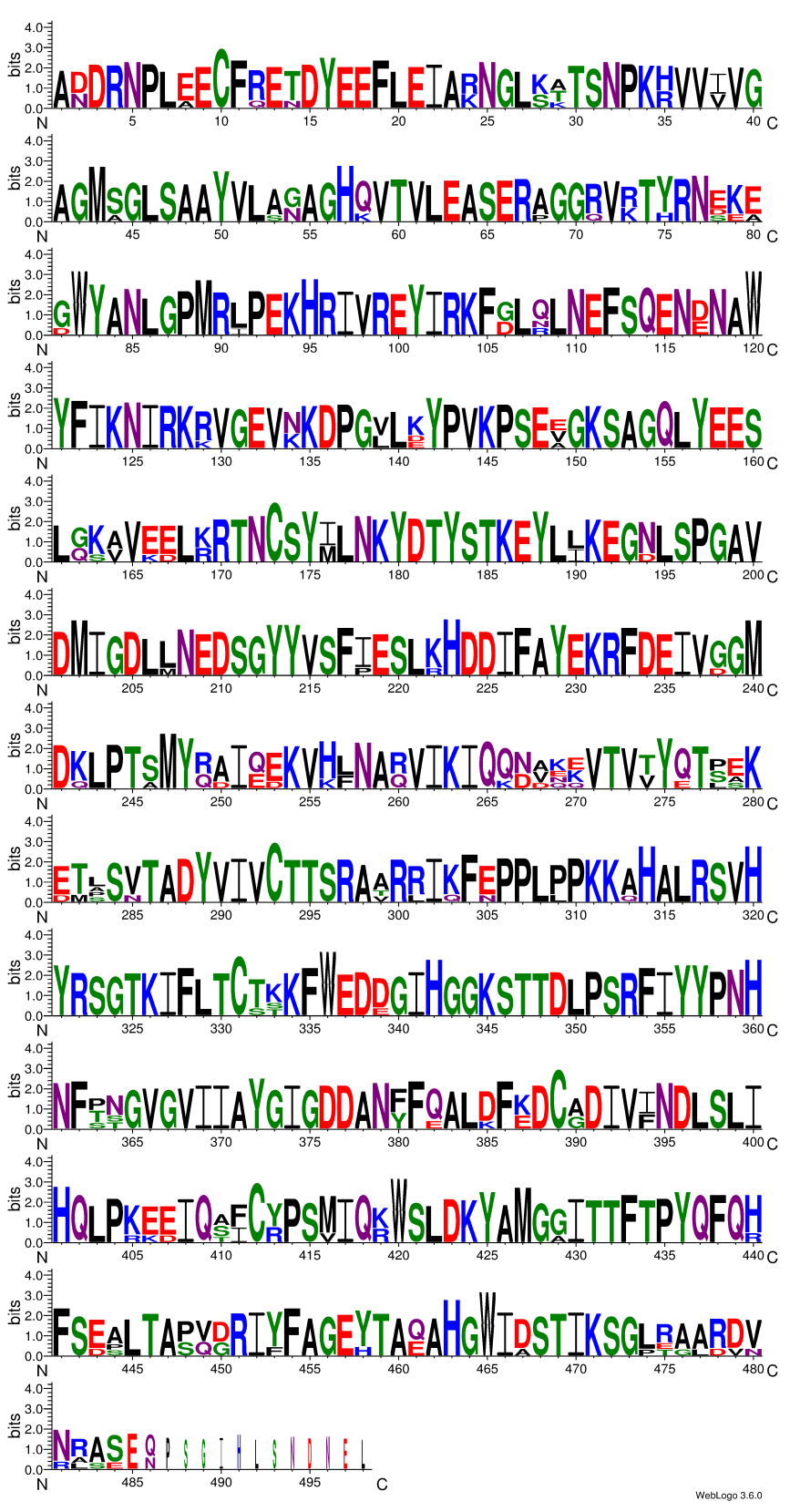
**

**Figure S2:** Sequence logo generated from the aligned sequence. The sequence logo displays the most conserved amino acid residues around the active/metal ion binding site from SV-LAAOs and their bacterial, fungal, plants and mammalian homologs.


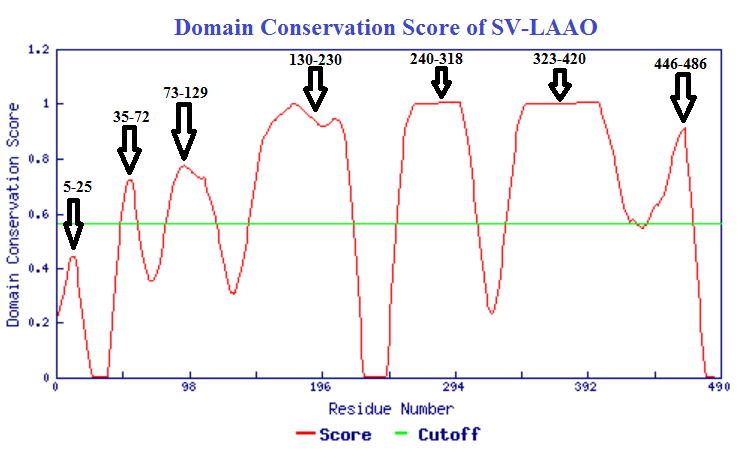


**Figure S3:** ThreaDom-based domain prediction in the domain conservation score profile. Seven individual domains (belonging to three main domains) are shown by arrows. The amino acid residues range have been shown above each domain.
